# Factors associated with negative colonoscopy in participants with a positive faecal immunochemical test from the Danish Colorectal Cancer Screening Program – a population‐based study

**DOI:** 10.1111/codi.16886

**Published:** 2024-01-31

**Authors:** Lea Østergaard Hansen, Mathias Benjamin Fürst, Thomas Bjørsum‐Meyer, Benedicte Schelde‐Olesen, Ulrik Deding, Lasse Kaalby

**Affiliations:** ^1^ Department of Surgery Odense University Hospital Odense Denmark; ^2^ Department of Clinical Research University of Southern Denmark Odense Denmark

**Keywords:** clean colon, colorectal cancer screening, negative colonoscopy, negative screening colonoscopy, personalized screening, risk factors

## Abstract

**Aim:**

In the Danish Colorectal Cancer Screening Program (DCCSP), 37% of participants undergoing colonoscopy have a negative result with no obvious findings that can be attributed to a positive faecal immunochemical test (FIT). The aim of this work was to identify predictors for a negative colonoscopy in DCCSP participants with a positive FIT.

**Method:**

We included 73 655 FIT‐positive DCCSP participants using the Danish Colorectal Cancer Screening Database and linked their screening results with data from several other national health registers. We stratified participants by all predictors, and compared them using multivariate logistic regression analysis. Results are reported as odds ratios (ORs).

**Results:**

We found that having a condition linked to gastrointestinal bleeding, for example fissures, haemorrhoids and inflammatory bowel disease, was strongly associated with the probability of having a negative colonoscopy [OR 2.77 (95% CI 2.59, 2.96)]. FIT concentration was inversely related to the probability of a negative colonoscopy, the OR decreased steadily from 0.79 (95% CI 0.75, 0.83) in the 40–59 μg/g group, to 0.44 (95% CI 0.42, 0.46) in the ≥200 μg/g group. Women had a 1.64 (95% CI 1.59, 1.70) times higher probability of a negative colonoscopy than men.

**Conclusion:**

Our findings indicate that baseline conditions linked to gastrointestinal bleeding are an associating factor with having a negative colonoscopy. The same is true for low FIT concentration and female sex. Further studies with similar findings could suggest that an incorporation of these factors into a personalized screening approach by differentiating between diagnostic modalities could improve the process for the participant while alleviating the health care system.

**What does this paper add to the literature?**

This study provides further knowledge about associations between patient characteristics and negative colonoscopy following a positive FIT in colorectal cancer screening participants, and includes patient‐related risk factors, which have been scarcely reported in previous literature. The knowledge obtained from this study therefore adds important data for more personalized screening in the future.

## INTRODUCTION

Colorectal cancer (CRC) is the second leading cause of cancer‐related death worldwide [1]. The stage at diagnosis is crucial to the prognosis, as the 5‐year survival rate drops from 93% for Stage I to 8% for Stage IV disease [2]. Furthermore, CRC develops slowly, rendering it a type of cancer prone to secondary prevention [3]. An increasing number of countries have adopted CRC screening programmes to detect precursor lesions or cancers at early stages [4]. In Denmark, people are screened for CRC by submitting a stool sample, which is analysed for faecal haemoglobin (f‐Hb) by a faecal immunochemical test (FIT). Participants are referred for colonoscopy if the FIT is positive (f‐Hb concentration ≥ 20 μg Hb/g faeces) [5]. However, in 37% of these colonoscopies, no neoplastic lesions are found that can explain the bleeding [6]. The occurrence of f‐Hb without any obvious source of bleeding has previously been investigated, but no single explanation is evident [7, 8, 9].

In a meta‐analysis from 2018, researchers found multiple risk factors, known at the time of FIT invitation, that were associated with false‐positive and false‐negative FIT results in CRC screening participants [10]. These included sex, age, smoking and use of different prescription medications such as antiplatelet agents and proton pump inhibitors. However, none of the included studies examined the effect of quantitative FIT concentrations, and only two studies evaluated socioeconomic factors, primarily education but not income. This leaves a significant gap in the understanding of what predicts a negative CRC screening outcome.

Therefore, we conducted a register‐based study with the aim of investigating the association between patient‐related risk factors, including socioeconomic factors, health conditions and FIT value, and negative colonoscopy in Danish CRC screening participants undergoing colonoscopy after a positive FIT.

## METHOD

We conducted a retrospective register‐based study on the participants of the Danish Colorectal Cancer Screening Program.

### Study population

We included all citizens participating in the first round of the Danish Colorectal Cancer Screening Program (DCCSP) between March 2014 and December 2017. The DCCSP was launched in 2014, and invites all Danish citizens aged 50–74 years to submit a stool sample for analysis. The sample kits contain an OC‐Auto Sampling Bottle 3 (Eiken Chemical Co, Japan) with 2.0 mL of buffer inside and a probe, which the participant uses to collect approximately 10 mg faeces. The sample kit is sent to participants' homes alongside an invitation letter, a pamphlet from the Danish Health Authority, an instruction on how to collect the stool sample and how to apply their ID label on the sample tube and finally a prepaid envelope for returning the sample tube by prioritized mail. The tests are sent to one of five regional medical laboratories in Denmark, where they are analysed for f‐Hb using the FIT (OC‐Sensor DIANA, Eiken Chemical Co, Tokyo, Japan), with the threshold in Denmark being 20 μg Hb/g faeces for a positive test [6]. These laboratories each cover all the FIT assays of their respective healthcare region and are ISO‐15189 accredited.

Participants with a positive FIT are invited for colonoscopy within 14 days. Before the procedure, the participant has to cleanse the bowel for optimal visualization of the colonic mucosa. Any histopathological results from the colonoscopy have to be given to the participant within 7 days [5]. A flow chart of the study population is shown in Figure 1.

**FIGURE 1:**
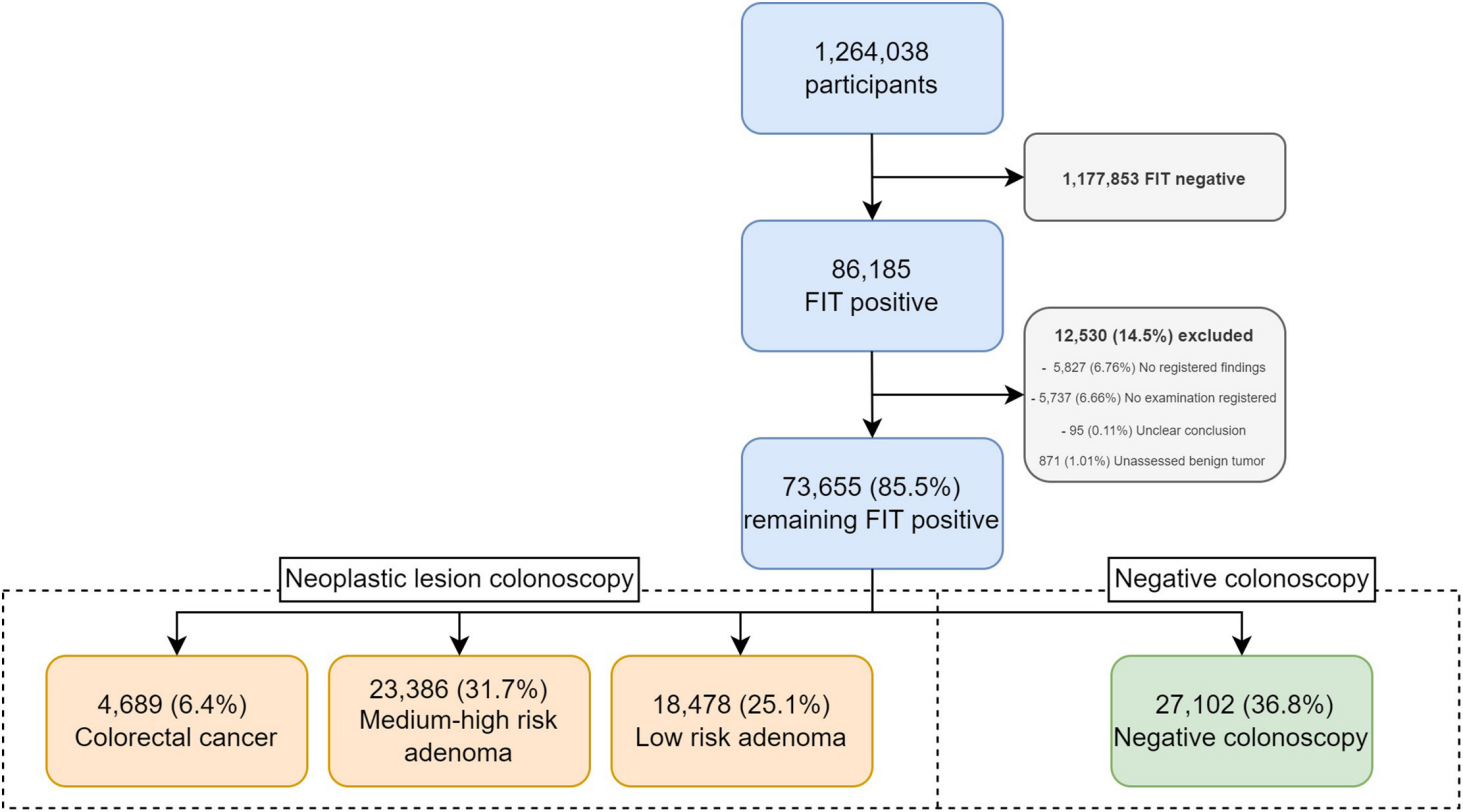
Participant flowchart.

### Data sources

Data were extracted from the Danish Colorectal Cancer Screening Database. Information on comorbidities was collected from the Danish National Patient Register [11]. A full list of included conditions and their ICD‐10 codes can be found in Table S1. We obtained data on the use of medication potentially causing gastrointestinal (GI) bleeding from the Danish National Prescription Register [12]. Data on socioeconomic status were extracted from the Income Statistics Register [13] and the Danish Educational Register [14]. Lastly, we used the Central Person Register (CPR) for information on CPR status (alive/deceased/emigrated) [15].

### Patient characteristics

Data from the different registers were included at baseline. Factors investigated in this study were: sex, age, FIT concentration, prescription medication linked to GI bleeding (Table S1), highest degree of education, annual income, conditions associated with GI bleeding (Table S1) and baseline comorbidities. Sex was defined as either male or female. Age was divided into the following groups: 50–54, 55–59, 60–69 and 70–75 years. FIT concentration was categorized as 20–39, 40–59, 60–99, 100–199 and ≥200 μg/g. Prescription medication was divided as follows: 0, 1, 2 or 3 (the number referring to how many different categories of relevant prescription medication a participant had redeemed up to 16 weeks prior to the FIT analysis date; i.e. a participant who redeemed two prescription within the 16 weeks, but from the same category, will therefore be in category 1, and likewise a participant who redeemed from two different categories would be categorized as 2). A full list of included medication groups and their ATC codes can be found in Table S1. The highest degree of completed education was divided into the following groups: elementary school, high school/vocational school, short–intermediate education (2–4 years) and long education (5–6 years). Annual income was defined as four within‐the‐sample quartiles, where group 1 was the lowest and group 4 the highest. We examined income on a 5‐year average, adjusted for inflation and the number of adults and children in the household. The 5 years went from baseline to 5 years in the past. Conditions suspected of causing GI bleeding were assessed up to 5 years prior to the FIT analysis date and defined as either ‘yes’ or ‘no’ in regard to having one or more of these conditions within 5 years of baseline. The Charlson Comorbidity Index (CCI) was used to measure the degree of comorbidity in patients, it is calculated by the standard method presented by Quan [16]. We expected existing comorbidities to affect the likelihood of having a negative colonoscopy after a positive FIT. For this reason, we applied a standard CCI, categorized as 0, 1, 2 or >2 [17].

### Outcomes of colonoscopy

The possible outcomes of a screening colonoscopy are CRC, high‐risk adenoma, medium‐risk adenoma, low‐risk adenoma and negative colonoscopy. If CRC is suspected during colonoscopy, an expedited diagnostic cancer pathway is initiated, and the final diagnosis is histologically verified. High‐risk adenoma is defined as either having an adenoma ≥20 mm or the presence of five or more adenomas regardless of size. Medium‐risk adenoma is defined with the presence of either an adenoma between 10 and 19 mm or three to four adenomas regardless of their size or tubule‐villous/villous morphology or an adenoma with high‐grade neoplasia. High‐risk adenomas and medium‐risk adenomas were combined for simplicity of interpretation. Low‐risk adenoma is defined as having fewer than three adenomas, all less than 10 mm. Low‐risk adenomas are always tubular in morphology and have low‐grade neoplasia histologically [18]. Negative colonoscopy is defined as a colonoscopy without any neoplastic findings. This means that CRC, high‐risk adenoma, medium‐risk adenoma and low‐risk adenoma are all ruled out. Citizens with negative colonoscopy enter an 8‐year quarantine from the screening programme and are invited again thereafter, provided that they are <75 years of age.

To achieve a valid outcome, only participants with complete colonoscopy were included. This means that the endoscope reached the caecum with good visibility throughout the colon.

### Statistics

For comparison of the study population groups, descriptive statistics were used. The chi‐square test was used to compare the groups on categorical variables. A logistic regression model was employed to compare the exposure groups on the odds of having a negative colonoscopy. Results are reported as both adjusted and crude odds ratios (ORs) with 95% CIs. Both univariate and multivariate logistic regression models were performed for all measured outcomes, the latter adjusting for confounders. The control groups were the ones with positive findings at colonoscopy. We excluded all participants with missing data on at least one predictor. All analyses were performed in Stata 17.0 [19].

### Ethics

We used pseudo‐anonymized data from all participants, which were stored on secure logged servers hosted by Statistics Denmark. The data will therefore not be available upon request, but are available to all researchers who apply for access. The study was registered with the regional archive of research projects handling personal information (journal no. 19/32137) in concordance with both Danish and European data protection legislation.

## RESULTS

### Characteristics

During the first round of CRC screening in Denmark 1 264 038 participants submitted an eligible stool sample. The vast majority (1 177 853) were FIT negative, leaving 86 185 FIT‐positive samples. Missing data and registry errors excluded 12 530 (14.54%) participants. The remaining 73 655 (85.36%) FIT‐positive participants were divided into categories based on colonoscopy result as shown in Figure 1: 27 102 (36.8%) participants had a negative colonoscopy, 4689 (6.37%) were found to have colorectal cancer and the remaining 14 864 (56.84%) had adenomas.

Observed differences for all patient characteristics are presented in Table 1. More men (56.85%) than women (43.15%) underwent colonoscopy, but the proportion of negative colonoscopies was higher in women (43.88%) than in men (31.42%). As age increased, the proportion of negative colonoscopies decreased significantly, with 6254 (51.43%) participants in the 50–54 years age group and 7013 (31.95%) participants in the 70–75 years age group having a negative colonoscopy.

**TABLE 1:** Characteristics of the study population by colonoscopy result.

	Negative colonoscopy (*N* = 27 102), *n* (%)	Neoplastic colonoscopy findings (*N* = 46 553), *n* (%)	Chi‐square test *p*‐value
Sex			
Female	13 945 (43.88)	17 838 (56.12)	<0.001
Male	13 157 (31.42)	28 715 (68.58)	
Age group (years)			
50–54	6254 (51.43)	5907 (48.57)	<0.001
55–59	4114 (40.41)	6067 (59.59)	
60–69	9721 (33.11)	19 639 (66.89)	
70–75	7013 (31.95)	14 940 (68.05)	
FIT concentration group (μg Hb/g faeces)			
20–39	11 623 (44.61)	14 434 (55.39)	<0.001
40–59	4669 (38.66)	7407 (61.34)	
60–99	3759 (35.85)	6726 (64.15)	
100–199	2940 (31.88)	6283 (68.12)	
≥200	4111 (26.00)	11 703 (74.00)	
Prescription medication			
0	10 634 (34.78)	19 941 (65.22)	<0.001
1	11 622 (37.80)	19 122 (62.20)	
2	4225 (39.08)	6587 (60.92)	
3	621 (40.75)	903 (59.25)	
Educational level			
Elementary school	7199 (35.66)	12 988 (64.34)	<0.001
High school/vocational school	12 431 (35.98)	22 114 (64.02)	
Short–intermediate	5483 (39.63)	8352 (60.37)	
Long	1488 (38.94)	2333 (61.06)	
Income quartile			
1st quartile	8153 (36.24)	14 347 (63.76)	0.117
2nd quartile	6988 (37.17)	11 811 (62.83)	
3rd quartile	6183 (36.95)	10 550 (63.05)	
4th quartile	5514 (36.85)	9450 (63.15)	
GI bleeding at baseline			
Yes	2438 (59.95)	1629 (40.05)	
No	24 664 (35.44)	44 924 (64.56)	
Region			
Northern Jutland	3710 (41.14)	5308 (58.86)	<0.001
Central Jutland	5609 (32.89)	11 445 (67.11)	
Southern Denmark	6846 (39.80)	10 357 (60.20)	
Capital	6833 (38.30)	11 010 (61.70)	
Zealand	4104 (32.74)	8433 (67.26)	
CPR status			
Alive	25 119 (92.68)	42 470 (91.23)	<0.001
Deceased	1905 (7.03)	3979 (8.55)	
Emigrated	78 (0.29)	104 (0.22)	
Charlson Comorbidity Index score			
0	20 426 (36.45)	35 617 (63.55)	0.001
1	2292 (38.16)	3714 (61.84)	
2	3287 (37.29)	5527 (62.71)	
>2	1097 (39.29)	1695 (60.71)	

Abbreviations: CPR, Central Person Register; FIT, faecal immunochemical test; GI, gastrointestinal; Hb, haemoglobin.

FIT concentration was inversely related to negative colonoscopy, with 11 623 (44.61%) participants in the lowest‐concentration group (20–39 μg Hb/g) having a negative colonoscopy and 4111 (26.00%) participants in the highest‐concentration group (≥200 μg Hb/g) having a negative colonoscopy. As the amount of concurrent prescription medication linked to GI bleeding increased, so did the proportion of negative colonoscopies, with 10 634 (34.78%) participants in group 0 having a negative colonoscopy, increasing to 621 (40.75%) participants in group 3. Having GI bleeding at baseline saw 2438 (59.95%) participants with a negative colonoscopy, whereas the absence of GI bleeding at baseline saw 24 664 (35.44%) participants with a negative colonoscopy. In total, 2792 (3.8%) participants scored >2 in the CCI, signifying extensive comorbidity.

### Factors for a negative colonoscopy

The results of our univariate and multivariate analyses are presented in Table 2.

**TABLE 2:** Factors influencing the risk of negative colonoscopy

	Univariate analysis		Multivariate analysis	
	OR (95% CI)	*p*‐value	OR (95% CI)	*p*‐value
Age group (years)				
50–54				
55–59	0.64 (0.61, 0.68)	<0.001	0.63 (0.59, 0.67)	<0.001
60–69	0.48 (0.45, 0.49)	<0.001	0.46 (0.44, 0.48)	<0.001
70–75	0.44 (0.42, 0.46)	<0.001	0.42 (0.40, 0.44)	<0.001
Sex				
Female	1.70 (1.66, 1.76)	<0.001	1.64 (1.59, 1.70)	<0.001
Male				
FIT concentration group (μg Hb/g faeces)				
20–39				
40–59	0.78 (0.75, 0.82)	<0.001	0.79 (0.75, 0.83)	<0.001
60–99	0.69 (0.66, 0.73)	<0.001	0.70 (0.66, 0.73)	<0.001
100–199	0.58 (0.55, 0.61)	<0.001	0.59 (0.56, 0.62)	<0.001
≥200	0.44 (0.42, 0.46)	<0.001	0.44 (0.42, 0.46)	<0.001
Income quartile				
1st quartile				
2nd quartile	1.04 (1.00, 1.08)	0.0490	0.98 (0.93, 1.04)	0.9380
3rd quartile	1.03 (0.99, 1.08)	0.1460	0.99 (0.95, 1.02)	0.2860
4th quartile	1.03 (0.98, 1.07)	0.2270	1.00 (0.95, 1.05)	0.9320
Educational level				
Elementary school				
High school/vocational school	1.01 (0.98, 1.05)	0.447	1.04 (1.00, 1.08)	0.0510
Short–intermediate	1.18 (1.13, 1.24)	<0.001	1.16 (1.11, 1.22)	<0.001
Long	1.15 (1.07, 1.24)	<0.001	1.23 (1.14, 1.33)	<0.001
Charlson Comorbidity Index score				
0				
1	1.08 (1.02, 1.14)	0.0090	1.05 (0.99, 1.11)	0.0140
2	1.04 (0.99, 1.09)	0.1250	1.11 (1.06, 1.17)	<0.001
>2	1.13 (1.04, 1.22)	0.0020	1.15 (1.06, 1.25)	0.0010
Baseline conditions causing GI bleeding				
Yes	2.73 (2.56, 2.91)	<0.001	2.77 (2.59, 2.96)	<0.001
No				
Prescription medication				
0				
1	1.14 (1.10, 1.18)	<0.001	1.21 (1.17, 1.25)	<0.001
2	1.20 (1.15, 1.26)	<0.001	1.36 (1.29, 1.43)	<0.001
3	1.29 (1.16, 1.43)	<0.001	1.46 (1.31, 1.63)	<0.001

Abbreviations: FIT, faecal immunochemical test; GI, gastrointestinal; Hb, Haemoglobin.

#### Age and sex

A tendency towards a decreasing probability of negative colonoscopy as age increased was observed, from an OR of 0.63 (95% CI 0.59, 0.67) in the 55–59 years age group to 0.42 (95% CI 0.40, 0.44) in the 70–75 years age group, when compared with the 50–54 years age group. A strong association was seen between female sex and the probability of negative colonoscopy, with a 1.64 (95% CI 1.59, 1.70) times higher probability of a negative colonoscopy compared with men.

#### FIT concentration

FIT concentration was inversely related to negative colonoscopy, as the OR of negative colonoscopy decreased steadily throughout the groups. The group with the lowest fit values (the 20–39 μg/g group) was the reference group, and the OR in the 40–59 μg/g group was 0.79 (95% CI 0.75, 0.83), decreasing to 0.70 (95% CI 0.66, 0.73) for the 60–99 μg/g group. There was further decrement in the 100–199 and ≥ 200 μg/g groups with ORs of 0.59 (95% CI 0.56, 0.62) and 0.44 (95% CI 0.42, 0.46), respectively.

#### Socioeconomic factors

An association was observed between negative colonoscopy and the two highest levels of education, with short–intermediate having an OR of 1.16 (95% CI 1.11, 1.22) and long an OR of 1.23 (95% CI 1.14, 1.33) when compared with elementary school. The association was, however, not found for high school/vocational school, as a *p*‐value of 0.051 and CI borders rendered the OR of 1.04 (95% CI 1.00, 1.08) nonsignificant. Income was not statistically significantly related to negative colonoscopy as the three top quartiles had ORs of 0.98 (95% CI 0.93, 1.04), 0.99 (95% CI 0.95, 1.02) and 1.00 (95% CI 0.95, 1.05) for quartiles 2, 3 and 4, respectively, when compared with income group 1.

#### Bleeding disorders, prescription medication and comorbidity

A strong association was observed between having a baseline condition causing GI bleeding and negative colonoscopy, as a person with a condition suspected of causing GI bleeding had a 2.77 (95% CI 2.59, 2.96) times higher probability of having a negative colonoscopy than a person without.

The OR for negative colonoscopy increased with the number of different prescription medications a participant was using, where 1 had an OR of 1.21 (95% CI 1.17, 1.25), rising to 1.36 (95% CI 1.29, 1.43) for 2, when compared with 0. The OR for group 3 was 1.46 (95% CI 1.31, 1.63), but due to overlapping CIs it could not, with certainty, be concluded that the OR for group 3 exceeded that for group 2.

Concerning CCI, significance was found for participants in groups 2 and >2 with ORs of 1.11 (95% CI 1.06, 1.17) and 1.15 (95% CI 1.06, 1.25), respectively, when compared with group 0. Having only one comorbidity did not show a significant correlation with negative colonoscopy.

The differences in ORs between multivariate and univariate analysis were minimal as shown in Table 2, except for analysis on prescription medication, which was more pronounced when adjusting for confounders in the multivariate analysis (Figure 2).

**FIGURE 2:**
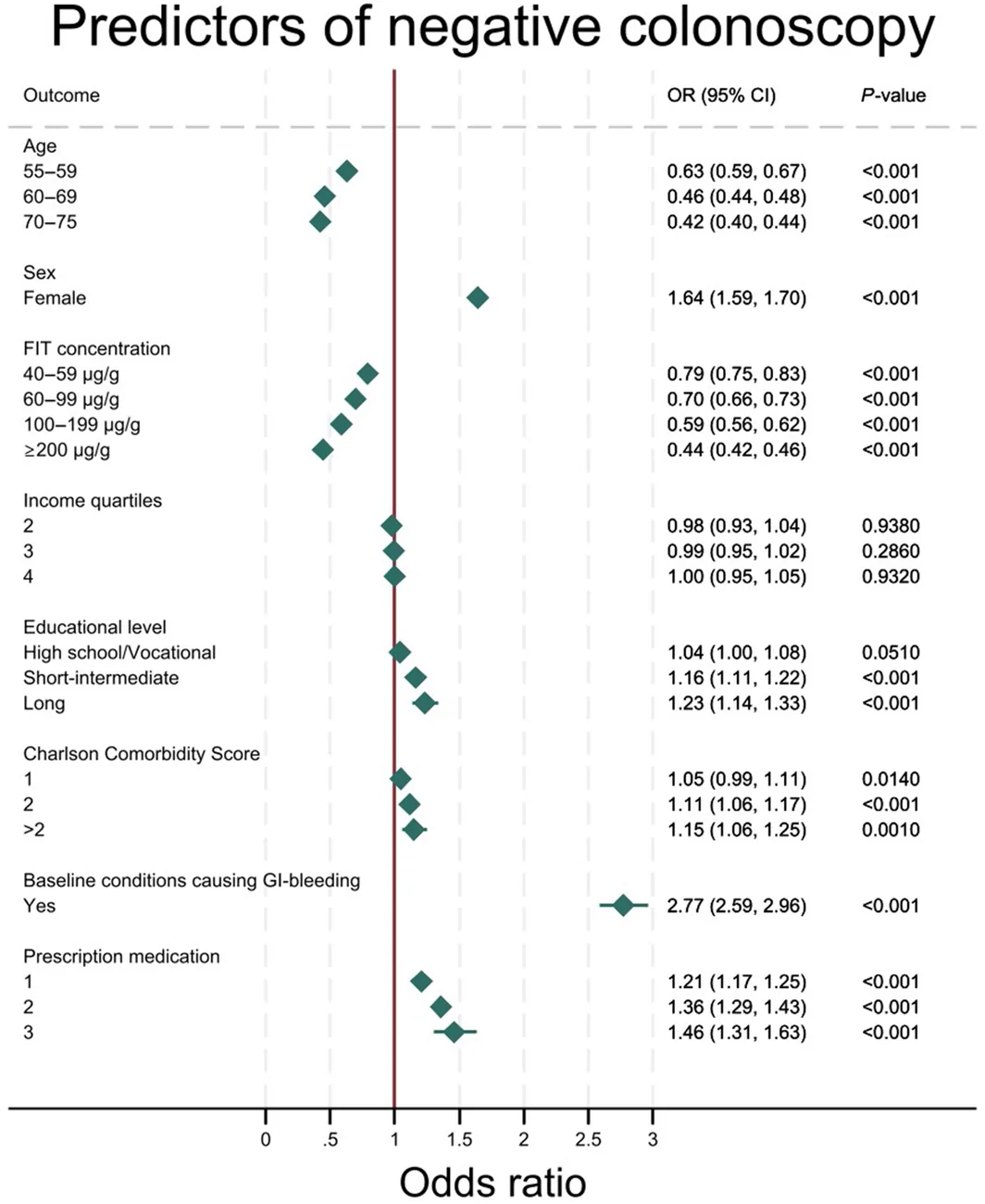
Multivariate analysis of predictors of negative colonoscopy.

## DISCUSSION

Our results show that several predictors are associated with the probability of having a negative colonoscopy. First, taking medication linked to GI bleeding and having a condition associated with GI bleeding were related to negative colonoscopy, particularly the latter with an OR of 2.77. This is not consistent with the findings in the meta‐analysis by de Klerk et al. that included 10 studies looking at anticoagulants, antithrombotics, aspirin and warfarin in association with false‐positive FIT [10]. Here, the authors found no significant risk for a false‐positive FIT in participants using anticoagulant medication. Our analysis shows that the risk of negative colonoscopy increases with the number of medications linked to GI bleeding. Some of the difference may be due to the difference in population size between the studies of the meta‐analysis versus the rather large population included in our study [10]. They did, however, find a significantly higher risk for a false‐positive FIT within patients using nonsteroidal anti‐inflammatory drugs. Including each of the different types of medication within in our study as a separate category in the multivariable regression model did not affect our results. A Spanish study from 2021 found an association between medications linked to GI bleeding and the risk of having a false‐positive FIT, much like the one shown in the results of this study [20]. Furthermore, they found an even stronger association between female sex and false‐positive FIT than in our study.

Our results suggest that women have a higher probability of having a negative colonoscopy than men (OR 1.64). This supports the results of multiple studies included in the meta‐analysis by de Klerk et al [10]. Five studies found that women had a significantly higher risk of having a negative colonoscopy following a positive FIT [21, 22, 23, 24, 25]. Some studies showed that younger women in particular are less likely to have findings during colonoscopy (polyps and adenomas) [26]. Using this logic, Sung et al. proposed in 2008 that women could enter the CRC screening programme later than men [27]. However, studies showed that right‐sided adenomas are more likely to be missed during colonoscopy [28, 29]. Right‐sided adenomas are also more common in women than men [30, 31]. This could suggest that women have a true positive FIT but a false‐negative colonoscopy with missed findings. This could be one explanation of our results and needs to be further explored, possibly by looking at interval cancers after negative colonoscopy, since this would then be higher for women. This has been explored in a study in which the authors looked at interval cancers following a negative FIT, and found that men had a higher risk of the event [32]. More nuance is needed to better understand this pattern, but the results support the notion that women with a positive FIT and a negative colonoscopy could have other, nonneoplastic explanations for their GI bleeding or missed colonoscopic findings.

Our results indicate that FIT concentration is inversely related to negative colonoscopy, so the higher the concentration the lower the probability of a negative colonoscopy. Previous studies have found that the average FIT value for women is generally lower than the FIT value for men [33]. When looking at our results from the univariate analysis of sex in association with a negative colonoscopy this notion seems to be supported. Since women more often have a negative colonoscopy but also more often have a lower FIT, future studies should investigate the association between female sex, FIT value and endoscopic findings compared with those of men, since this could set the ground for more personalized screening strategies.

The association between negative colonoscopy and both FIT concentration and comorbidity is interesting because several studies have shown an association between f‐Hb and several diseases seemingly unrelated to CRC [7, 8, 34, 35, 36]. The hypothesis is that these diseases have a systemic (potentially inflammatory) component that affects the bowel and facilitates lower GI bleeding. This in turn might result in a positive FIT without any discernible neoplastic findings [7]. By this logic, f‐Hb may be a biomarker for future non‐CRC conditions. Our findings seem to be in support of the notion that something other than colorectal neoplasia causes the elevated f‐Hb concentration. The mechanics, however, have yet to be clearly established. This promotes the need for further studies on the disease patterns of participants with a negative colonoscopy but a positive stool sample.

When combining the observations of other studies with our findings, it appears that screening participants with a negative colonoscopy outcome seem to have a number of common and identifiable traits. Cementing the presented associations will be important for future initiatives that seek to address this high rate of negative colonoscopies to reduce the endoscopic burden both on and of this group of participants. One approach could be to optimize the CRC screening pathways through personalization that considers a wide range of factors associated with negative colonoscopy, and offer this group of patients a different investigation modality, for example CT colonography or camera capsule endoscopy. Additionally, these findings may appease patients by providing them with an answer for why their FIT was positive when no pathology was identified at colonoscopy.

### Strengths and limitations

The strengths of our study include the substantial population analysed, the individual‐level adjustments for confounding effects and that this is the first study to examine quantitative FIT concentrations and their association with negative colonoscopy. The study is limited by not having access to information on different lifestyle factors such as body mass index, smoking, exercise and diet. Another potential limitation is the lack of access to information about diseases from outside the hospital sector. We know that some diseases are primarily diagnosed and managed in the general practice sector, and are therefore not necessarily registered in the national databases used in this study. This could have affected our results to some degree. In particular, conditions that would not normally result in a hospital contact, such as haemorrhoids, may be underreported. It is also plausible that some negative colonoscopies are subject to misclassification, due to lesions being missed during investigation. These could especially concern the right‐sided adenomas that are more often missed during colonoscopy [28, 29]. Furthermore, the use of certain medications (such as anticoagulants or antiplatelet agents) is known to be a risk factor for GI bleeding, and since these are available over the counter in Denmark the actual number of citizens using medication linked to GI bleeding could be underreported [37, 38].

## CONCLUSIONS

Our study is the first to investigate different patient characteristic including FIT in association with negative colonoscopy in a substantial population. Our results show that FIT concentration, sex, age, prescription medication and having a condition linked to GI bleeding at baseline were all associated with the probability of a negative colonoscopy. While these associations still need to be clearly established, it seems likely that future initiatives attempting to personalize screening should take several of the identified factors into consideration.

## AUTHOR CONTRIBUTIONS

**Lea Østergaard Hansen:** Visualization; writing – original draft; writing – review and editing. **Mathias Benjamin Fürst:** Conceptualization; methodology; writing – original draft; investigation. **Thomas Bjørsum‐Meyer:** Conceptualization; supervision; writing – review and editing; project administration; visualization. **Benedicte Schelde‐Olesen:** Writing – review and editing; supervision; data curation; investigation; formal analysis; visualization. **Ulrik Deding:** Writing – review and editing; data curation. **Lasse Kaalby:** Conceptualization; methodology; data curation; investigation; validation; formal analysis; supervision; project administration; writing – review and editing; visualization.

## FUNDING INFORMATION

No funding has been received for this study.

## CONFLICT OF INTEREST STATEMENT

The authors of this study declare no conflict of interest.

## ETHICS APPROVAL AND CONSENT TO PARTICIPATE

Regional archive of research projects handling personal information (journal no. 19/32137).

## PERMISSION TO REPRODUCE MATERIAL FROM OTHER SOURCES

N/A.

## Supporting information


Table S1.

## Data Availability

The data used in this study was obtained from multiple registers, accessed via secure servers of Statistics Denmark. For this reason, we are not able to make it available, but others will be able to apply for access to the same raw data through Statistics Denmark, given the proper clearances.
