# Supplementary material for: Factors associated with negative colonoscopy in participants with a positive faecal immunochemical test from the Danish Colorectal Cancer Screening Program – a population‐based study
Source: Colorectal Dis. 2024 Jan 31;26(3):476–85. doi: 10.1111/codi.16886 (PMC13696433; doi:10.1111/codi.16886)
Supplement: Supplementary file 1 [file CODI-26-476-s001.docx]

Table S1

# Additional file 1

**Table S1. Conditions and medications suspected of causing gastrointestinal bleeding**

| **Conditions** | **ICD-10 classification** |
| --- | --- |
| Hemorrhage of anus and rectum | K625-K626 |
| Inflammatory Bowel Disease | K50-K52 |
| Diverticular disease | K57 |
| Hemorrhoids | I84 |
| Colorectal fissures | K60 |
| Gastrointestinal ulcers | K25-K28 |
| Gastritis | K29 |
|  | |
| **Medications** | **ATC code** |
| All Antiplatelet agents, parenteral and oral anticoagulants | B01A |
| Corticosteroids | H02, A07EA |
| All non-steroidal anti-inflammatory drugs | M01A |

**Abbreviations:** ICD, International Classification of Disease; ATC, Anatomical Therapeutic Chemical.
